# An overview of the role of chemokine CX3CL1 (Fractalkine) and CX3C chemokine receptor 1 in systemic sclerosis

**DOI:** 10.1002/iid3.70034

**Published:** 2024-10-11

**Authors:** Fatemehsadat Pezeshkian, Reza Shahriarirad, Hadiseh Mahram

**Affiliations:** ^1^ Shiraz University of Medical Sciences Shiraz Iran; ^2^ Thoracic and Vascular Surgery Research Center Shiraz University of Medical Sciences Shiraz Iran

**Keywords:** CX3CL1 protein, chemokine CX3CL1, cytokines, endothelial cells, rheumatic diseases, scleroderma

## Abstract

**Introduction:**

Systemic sclerosis (SSc) is a complex autoimmune disease characterized by fibrosis, vascular damage, and immune dysregulation. Fractalkine or chemokine (C‐X3‐C motif) ligand 1 (CX3CL1), a chemokine and adhesion molecule, along with its receptor CX3CR1, have been implicated in the inflammatory processes of SSc. CX3CL1 functions as both a chemoattractant and an adhesion molecule, guiding immune cell trafficking. This systematic review examines the role of CX3CL1 and its receptor CX3CR1 in the pathogenesis of SSc, with a focus on pulmonary and vascular complications.

**Methods:**

A systematic literature search was conducted across databases including PubMed, Scopus, and Web of Science from inception to November 2020. The search focused on studies investigating the CX3CL1/CX3CR1 axis in the context of SSc.

**Results:**

The review identified elevated CX3CL1 expression in SSc patients, particularly in the skin and lungs, where CX3CR1 is expressed on infiltrating immune cells. Higher levels of CX3CL1 were correlated with the severity of interstitial lung disease in SSc patients, indicating a potential predictive marker for disease progression. CX3CR1‐positive monocytes and NK cells were recruited to inflamed tissues, contributing to fibrosis and tissue damage. Animal studies showed that inhibition of the CX3CL1/CX3CR1 axis reduced fibrosis and improved vascular function.

**Conclusion:**

The CX3CL1/CX3CR1 axis plays a key role in immune cell recruitment and fibrosis in SSc. Elevated CX3CL1 levels are associated with lung and vascular complications, making it a potential biomarker for disease progression and a promising therapeutic target.

## Graphical abstract

This review investigates the role of fractalkine (FKN) and its receptor, CX3CR1, in systemic sclerosis (SSc) pathogenesis. Elevated levels of CX3CL1 in SSc correlate with interstitial lung disease progression, indicating its potential as a serological marker for skin and pulmonary complications in SSc patients.

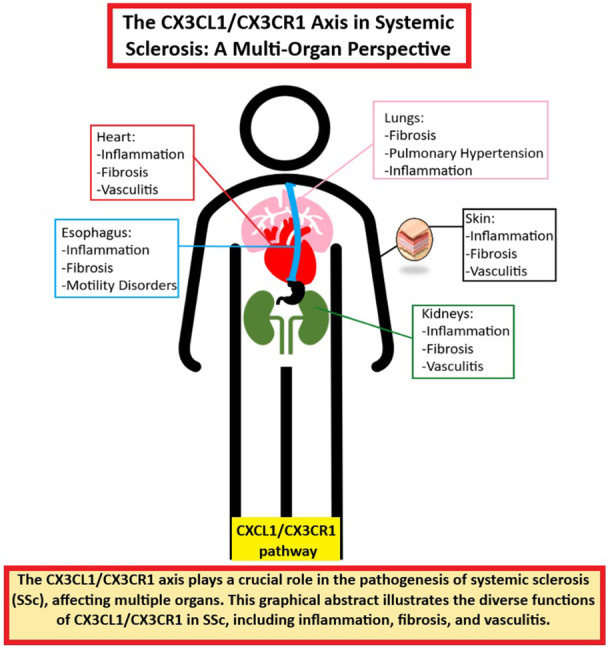

## 1 BACKGROUND

Systemic sclerosis (scleroderma, SSc) is a chronic labyrinthine systemic and autoimmune rheumatic connective tissue disorder distinguished by fibrosis, a hallmark of the disease, and vascular injury and involving multiple internal organs impacting morbidity and mortality.^1^, ^2^, ^3^ Generally, SSc presents with immoderate vascular damage, immunological activation, and collagen deposition, which are clinically interconnected.^4^, ^5^, ^6^, ^7^ Structural variability, along with Reynaud's phenomenon, a progressive insufficiency in vasodilatory competence of the vessels, makes up the vascular clinical manifestations.^8^ Scleroderma has two major manifestations: limited scleroderma, which causes cutaneous presentation typically in the face, hands, and arms, and diffuse SSc, involving substantial parts of the skin and internal organs such as the lungs, heart, kidneys, and esophagus.^9^

SSc's principal development is, in all likelihood, due to the unusual collection of extracellular matrix (ECM) constituents (i.e., collagen type I and III) with progressive loss of microvascular bed. However, the exact etiology and pathogenesis remain unclear. Many studies declared that cytokine secretion from fibroblasts, endothelial cells, inflammatory cells, and other cell kinds in the implicated organs plays a vital role in initiating and maintaining fibrosis. The crosstalk between activated fibroblasts, immune effectors, and endothelial cells is the primary culprit of the disease process.^10^ Skin biopsy specimens containing perivascular infiltrate of immune cells and macrophages are witnesses to their significance in disease institutions. Infiltrated T cells gravitate toward a phenotype of T helper type 2 (Th2) cells and release copious amounts of profibrotic cytokines, consisting of IL‐4, IL‐6, and IL‐13.^11^

Activated macrophages are the primary immune cells recruited in the fibrosis process and are considered influential in this process.^12^ Another event that is seen in patients with SSc is impaired neovascularization. The latest research proposes that neovascularization impairment could be connected to vasculogenesis and angiogenesis failure in the scleroderma. Remarkably, despite a significant elevation in plenty of angiogenic factors either in the serum or skin of SSc patients, compensatory angiogenesis does not happen conventionally.^13^

The blood vessel is the chief objective for the local immune activation and fibrosis initiation and propagation.^14^ Morphological alterations in the vessels vary from significant initial derangement with capillary thrombosis to frequently ineffective reparative neoangiogenesis with atypical capillary multiplication and nearly complete vessel loss with continuing ischemic injury in targeted tissues.^15^, ^16^ Endothelial disruption occurs due to an imbalance between lesion induction and repair courses that are noninvasive endothelium‐derived biomarkers.^17^, ^18^ Moreover, several soluble inflammatory endothelial mediators, like soluble fractalkine (sFKN) and endothelin‐1, accompany the pathogenesis of SSc.^19^, ^20^

**Figure 1:**
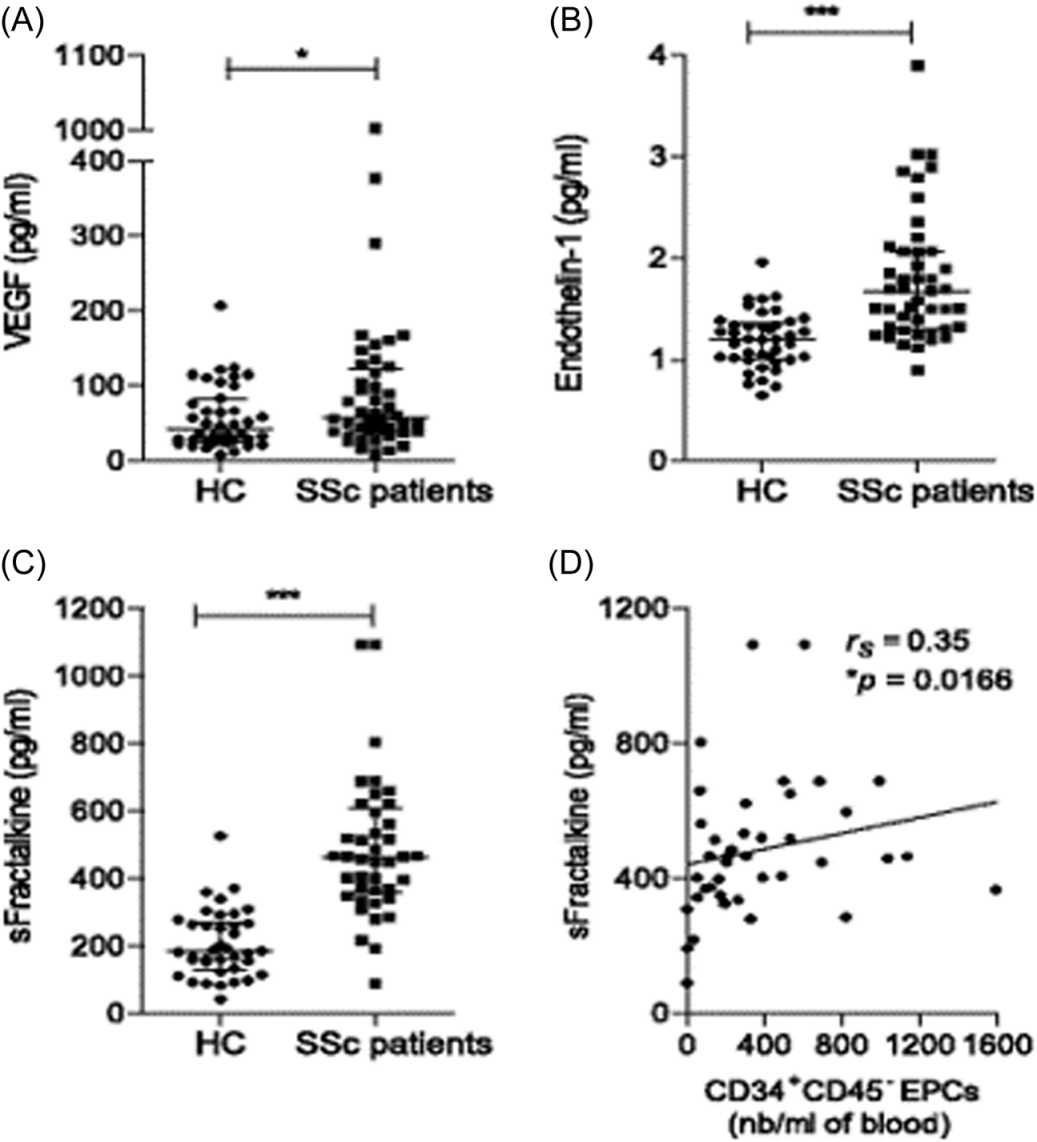
Soluble endothelial biomarker concentrations in patients with systemic sclerosis (SSc) compared with healthy control subjects (HC). (A) Vascular endothelial growth factor (VEGF). (B) Endothelin‐1. (C) Soluble fractalkine (s‐fractalkine). (D) Correlation between s‐fractalkine and CD34^+^CD45^−^ endothelial progenitor cell (EPC) counts (nb). Figure and values retrieved from a study by Benyamine et al. under the terms of the Creative Commons Attribution 4.0 International License (http://creativecommons.org/licenses/by/4.0/).^26^

Fractalkine (FKN) or chemokine (C‐X3‐C motif) ligand 1 (CX3CL1) is an unremarkable chemokine with two isoforms. The membrane‐bound one can be released by metalloprotease cleavage to form soluble isoform (sFKN), which acts not only as a chemoattractant but also as an adhesion molecule that is expressed on Pro‐inflammatory cytokines that are activated on endothelial cells.^21^, ^22^ Both adhesion and chemotaxis activity are mediated through G protein‐coupled receptor (GPCRG) CX3CR1.^23^, ^24^ CX3CR1 is an FKN receptor expressed on natural killer (NK) cells, cytotoxic effector T cells, and mature monocytes. FKN was expressed strongly on endothelial cells in inflammatory contexts of vascular injuries of lung tissues and affected skin in patients with SSc.^20^, ^21^ Enthrallingly, different genetic polymorphisms in the CX3CR1 gene, including the T280M and V249I, were seen in people with diffuse cutaneous SSC, and it could impact the expression and function of CX3CR1. Additionally, CX3CR1 polymorphism was also seen in association with pulmonary arterial hypertension.^25^ Furthermore, the levels of sFKN were notably raised in serum and were connected with digital ischemia existence and severe pulmonary fibrosis in those with diffuse cutaneous SSc (Figure 1).^20^, ^27^ Serum sFKN, a vigorous mediator of vasculopathy, corresponds to a very relevant target utilized in interventions regarding vascular features in SSc.^28^, ^29^ The high number of CX3CR1‐expressing cells in lung tissues of SSc patients and lesional skins of those with diffuse cutaneous involvement correlated considerably with the severity of pulmonary fibrosis and erythrocyte sedimentation rates.^30^, ^31^ Furthermore, it has been reported that SSc patients exhibit dysbiosis in their gut microbiota.^32^ Research indicates that the CX3CL1/CX3CR1 signaling pathway plays a crucial role in preserving gut integrity by eliminating pathogenic bacteria from the intestine.^33^ However, overactivation of this pathway can promote immune cell migration to peripheral tissues, resulting in significant tissue damage.^34^ Collectively, these data propose a substantial place for FKN as a mediator in SSc.

**Figure 2:**
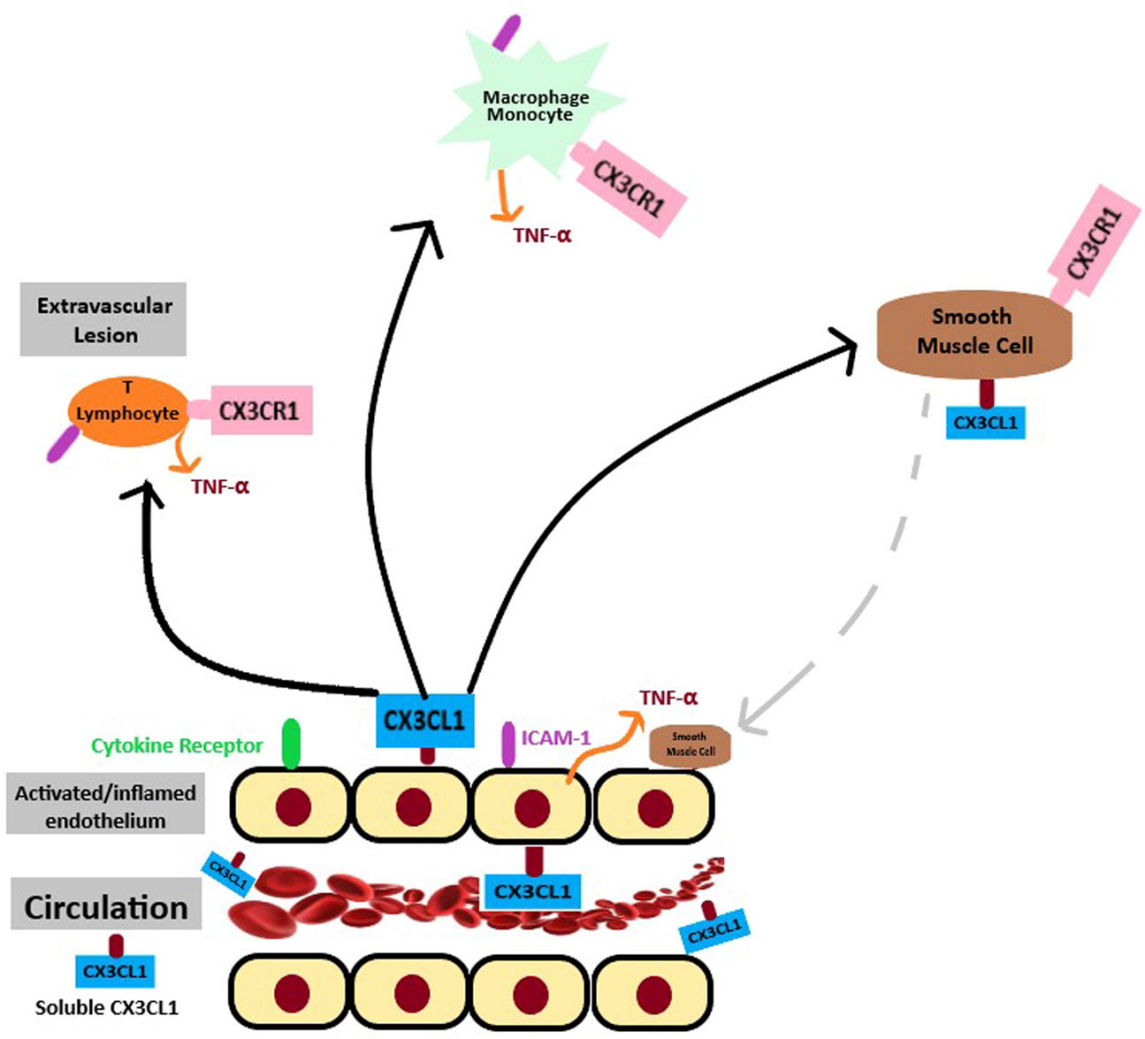
The CX3CL1/CX3CR1 pathway in vascular inflammation. Dysregulation of CX3CL1/CX3CR1 expression, activation of endothelial cells (ECs), and the migration of CD8^+^ T lymphocytes, CD4^+^ Th1‐type lymphocytes, macrophages, and smooth muscle cells (SMCs) all play critical roles in vasculopathy.

The recognition of the FKN/CX3CR1 pathway in SSc pathogenesis provides a novel outlook for targeted therapy that may limit immune‐mediated vascular injury and inflammatory fibrosis (Figure 2).^10^

In summary, this review aims to highlight the function of CX3CR1 and CX3CL1 (FKN) in systemic sclerosis.

## 2 CXC CHEMOKINES AND THEIR RECEPTORS

In the beginning, chemokines were labeled as chemoattractant cytokines produced at inflammation sites by various cell types of both nonhematopoietic and hematopoietic origin and are principal regulatory proteins for leukocyte trafficking and recruitment. Over 50 chemokines have been established by this date. They are intersected into four subfamilies, CX3C‐, CXC‐, C‐ and CC‐chemokine, based on the spacing and the number of the first two cysteines of the preserved cysteine's structural concept.^12^, ^35^, ^36^ Each chemokine class is prone to display various ranges of leukocyte specificity, and the created chemokines throughout the inflammatory process are presumed to arrange the quality, period, and extent of the cellular infiltration.^37^, ^38^, ^39^

The FKN is a membrane‐bound chemokine that acts both as an adhesion molecule and a chemoattractant and is expressed on endothelial cells that are Pro‐inflammatory cytokine activated. Overproduction of inducible nitric oxide synthase (iNOS), Interleukin‐1 (IL‐1), Tumor necrosis factor (TNF), and interleukin‐6 (IL‐6) is restrained by CX3CL1 signaling.^40^, ^41^, ^42^ In SSc patients, FKN is expressed on endothelial cells in lung tissues and infected skin. CX3CR1, The FKN receptor, is expressed on NK cells, cytotoxic effector T cells, and mature monocytes.^30^, ^31^ Interestingly, expression of CX3CR1 was observed to decline in NK cells of those with SSC.^10^ Neuroprotective signaling from motor neuron (MN) to microglia can be arbitrated through CX3CR1.^43^, ^44^ In the murine model of experimental autoimmune myositis, CX3CR1/FKN appears to be expressed on the infiltrated inflammatory cells. The interlinkage of CX3CL1 and its receptor, CX3CR1, could be a factor in cell migration as macrophages and cytotoxic T cells seize muscle tissues in idiopathic myelofibrosis. Elevated tissue and serum expression of CX3CR1 and CX3CL1 can be observed in rheumatoid arthritis (RA) and Sjogren's syndrome, affirming this chemokine participation in recruiting inflammatory cells in target organs.^45^, ^46^, ^47^

## 3 STRUCTURE, EXPRESSION, AND FUNCTION OF CX3CL1 AND CX3CR1

Bazan et al.^48^ made a significant contribution to the field of chemokine research by unveiling the discovery of a distinctive chemokine subclass, designated as CX3C. This subclass is exemplified by a solitary member known as CX3CL1 or FKN.^48^ FKN, a large protein consisting of 373 amino acids, is synthesized as a transmembrane molecule that encompasses a short cytoplasmic tail, an extracellular NH2‐terminal domain, a mucin‐like stalk, and a transmembrane α helix.^22^, ^49^, ^50^

CX3CL1 exhibits a unique structural organization, comprising a transmembrane domain and a soluble chemokine domain, and it is prominently expressed on the surface of a diverse array of cell types.^51^ These include vascular endothelial cells activated by TNF‐α, IFN‐c, or IL‐1, as well as neurons, fibroblasts, epithelial cells, macrophages, osteoblasts, and vascular smooth muscle cells.^22^, ^51^, ^52^, ^53^, ^54^, ^55^ Furthermore, CX3CL1 expression can be detected in various organs, such as the brain, skin, kidneys, and tonsils.^22^

The receptor for CX3CL1, known as CX3CR1, belongs to the G protein‐coupled receptor (GPCR) family and is expressed on select subsets of immune cells, including cytotoxic effector lymphocytes, NK cells, cytotoxic T cells (CTLs), B cells, Th1 cells, γδ T cells, dendritic cells, and nonclassical and intermediate monocyte subsets.^55^, ^56^, ^57^, ^58^ Notably, effector‐type lymphocytes expressing CX3CR1 display a cytotoxic phenotype characterized by the expression of granzyme B, TNF‐α, IFN‐γ, and perforin.^57^, ^58^

Interestingly, FKN possesses a distinctive structural configuration, existing in both membrane‐bound and soluble forms, endowing it with dual functionality as both a chemokine and an adhesion molecule.^51^ The membrane‐bound form of FKN has been detected in various cell types, including endothelial cells, astrocytes, neurons, osteoblasts, intestinal epithelium, and fibroblast‐like synoviocytes.^51^ Inflammatory cytokines such as interferon (IFN)‐γ, IL‐1, and TNF‐α strongly activate the membrane‐bound form of FKN, specifically on primary endothelial cells.^59^, ^60^

Moreover, the interaction between membrane‐bound CX3CL1 and its receptor CX3CR1 leads to robust adhesion of NK cells, monocytes/macrophages, and a specific subset of T cells to endothelial cells expressing FKN.^55^, ^61^ This adhesive interaction is of paramount importance in leukocyte trafficking and adhesion at the endothelium, where both chemotactic factors and adhesion molecules play crucial roles.^31^

A notable feature of the adhesion mediated by membrane‐bound FKN is its reliance solely on the binding affinity with CX3CR1, obviating the need for selectin‐mediated activation or integrin rolling.^50^, ^54^, ^61^ This unique CX3CL1‐CX3CR1 interaction‐based adhesion is remarkably stable, although it can be disrupted by metalloprotease‐induced cleavage.^62^ Moreover, membrane‐bound FKN promotes integrin activation via CX3CR1, leading to enhanced cell adhesion between integrins and fibronectin or intercellular adhesion molecule‐1 (ICAM‐1).^63^ In fact, cells expressing CX3CR1 attach more rapidly to immobilized FKN compared to vascular cell adhesion molecule‐1 (VCAM‐1), even without the requirement of inflow and cell tethering conditions.^64^ Consequently, FKN plays a vital role in facilitating the extravasation of circulating leukocytes by promoting cell adhesion during the tethering and transmigration steps.^50^

Furthermore, the co‐expression of CX3CR1 and integrin ligands, such as VCAM‐1 and ICAM‐1, results in significantly augmented cell adhesion compared to either system alone.^50^, ^65^ This suggests a synergistic effect of CX3CR1 and integrins in facilitating cell adhesion mediated by FKN.^50^, ^65^ In conclusion, the mechanism of cell adhesion mediated by membrane‐bound FKN and CX3CR1 appears to be more intricately orchestrated compared to conventional cell adhesion processes.^21^, ^22^, ^55^

In addition to its membrane‐bound form, FKN also exists in a soluble configuration generated through proteolytic cleavage, primarily by enzymes such as a disintegrin and metalloprotease (ADAM)‐10 or ADAM‐17, also called TNF‐ α‐converting enzyme (TACE).^59^, ^60^ Extensive investigations have shed light on the proteolytic shedding of FKN, revealing the involvement of ADAM17/TACE in response to cell activation and suggesting a potentially more prominent function for ADAM10 in the constitutive shedding role.^62^, ^66^

Soluble FKN, which is abundantly present at inflamed sites, serves as a chemoattractant for T cells, monocytes, and NK cells and plays a crucial role in directing migration of inflammatory cells expressing CX3CR1 upon attachment to endothelial cells.^59^, ^60^ Additionally, the proteolytic cleavage of FKN is tightly regulated and prominently enhanced under inflammatory conditions.^67^ Consequently, blocking the FKN‐CX3CR1 axis offers a promising strategy to prevent or attenuate inflammation by inhibiting FKN‐mediated cell adhesion and the migration of inflammatory cells.^68^

### 3.1 CX3CL1/CX3CR1 in monocytes/macrophages in systemic sclerosis

It has been revealed that FKN influences monocyte migration, and CX3CR1's expression has been reported on CD14^+^ monocytes.^22^, ^55^ There are three classes of peripheral blood monocyte subsets: CD14^+^ CD16^+ +^, which is nonclassical monocytes; CD14^+ +^ CD16^+^, which is intermediate monocytes; and CD14^+ +^ CD16^−^ which is classical monocytes. Intermediate and nonclassical monocytes significantly express CX3CR1.^69^, ^70^, ^71^ Intermediate and nonclassical monocytes play a role in tissue homeostasis and inflammation. Patrolling nonclassical monocytes controls vascular integrity during inflammatory and steady‐state situations, and intermediate monocytes express keen phagocytotic activity and produce profuse amounts of pro‐inflammatory cytokines in diverse inflammatory disorders.^72^ Human circulating FKN indicates proficient chemotactic activity for NK cells, macrophages/monocytes, and T cells that express CX3CR1.^22^, ^55^ Research showed that the CX3CL1–CX3CR1 axis might lead to inflammatory diseases and vascular injury through the recruitment of activated leucocytes.^21^, ^73^

SSc patients were observed to have rising serum levels of sFKN and its receptor CX3CR1 T cells and peripheral macrophages/monocytes, which pathologically enrolls monocytes into the skin of patients with SSc, arbitrating the institution and propagation of skin sclerosis. The quantity of cells exhibiting CX3CR1 is also raised in the lung tissues of patients with SSc.^74^, ^75^, ^76^

Although the role of FKN in the pathogenesis of lung fibrosis is unclear, a study in mice model revealed that bleomycin (BLC)‐induced lung fibrosis is associated with upregulation of CX3CR1 on fibroblasts and M2‐type macrophages, which play a critical role in fibrosis.^77^

The contribution of FKN to macrophage existence has been observed in humans as well as in murine models. In murine models with CX3CR1 deficiency, compromised survival of macrophages has been established under a number of conditions, from atherosclerosis to homeostasis to liver inflammation. FKN restrains the apoptosis of CX3CR1‐expressing macrophages through Akt activation in a PI3‐kinase‐dependent figure.^78^, ^79^

According to recent discoveries, FKN aids in regulating cytotoxic cells' recruitment to the inflamed endothelium. As a result, FKN could obtain an essential function in cytotoxic cell‐mediated endothelium damage and, as a consequence, vascular injury.^29^

### 3.2 CX3CL1/CX3CR1 in endothelial cells of systemic sclerosis

Pro‐inflammatory mediators induce the expression of CX3CL1 on endothelial cells, and it was found that the expression of CX3CL1 is increased in inflammatory vascular lesions.^80^, ^81^

FKN is one of the most influential molecules that monitor the trafficking of inflammatory cells through the endothelium. Hasegawa et al. discovered pronounced expression levels of CX3CR1 among inflammatory cells, colocalizing with endothelium expression of CX3CL1 in objective organs like skin and lung of patients with SSc.^20^ Another study pointed out the increased levels of circulating FKN and endothelial microparticles (EMPs) as a sign of activation of the endothelial inflammation and homeostasis disruption in patients with SSc that could single out its potential value as a biomarker of disease severity and organ involvement.^20^, ^82^

A positive association among endothelial progenitor cell (EPC) mobilization and levels of circulating FKN in a setting of endothelial activation in patients with SSc recommended that FKN, as an angiogenic chemokine, activates the proliferation and migration of endothelial cells, migration of EPCs, and in vitro formation of a tube‐like structure and stimulates the in vivo formation of new blood vessels even in ischemic disease.^13^, ^82^, ^83^, ^84^

Advancement of in vivo imaging technology uncovered the distinct behavior of CX3CR1‐expressing monocytes on vascular endothelial cells.^85^, ^86^ CX3CR1‐expressing monocytes are named patrolling monocytes to guard the luminal side of resting endothelial cells to control vascular abnormalities.

Under steady conditions, this attachment is mainly affiliated with FKN and lymphocyte function‐associated antigen‐1 (LFA‐1).^85^ Nonetheless, the moment endothelial cells get activated due to damage or inflammation, FKN is impelled on the luminal side, and patrolling monocytes steadily attaches to the vascular endothelium via the interlinkage of FKN and CX3CR1.^12^, ^86^ These CX3CR1‐expressing monocytes attach to begin local inflammation through other chemokines and cytokine production, which activates and recruits other neutrophils or inflammatory monocytes at the site.^86^ Therefore, the FKN and CX3CR1 interlinkage is a methodical mechanism for the surveillance of endothelial abnormalities in tissues and the swift recruitment of leukocytes to escalate inflammatory reactions.^68^

Observations in NK cells exhibited a decreased expression of CX3CR1 that could indicate its association with membrane‐bound endothelial FKN and the chemoattracted NK cells recruitment via sFKN in the direction of the organ through inflamed endothelium.^20^ On the other hand, a study by Kasama et al. reported upregulated CX3CR1 expression in endothelial cells and the cumulation of activated inflammatory cells, which could result in pathophysiologic events causing skin vasculitis.^29^, ^87^

The expression of CX3CR1 is a characteristic of NK cells retaining inflated levels of perforin and intracellular granzyme B.^58^, ^88^ Due to the fact that these two substances are recognized to be in charge of endothelial injury in cell‐mediated cytotoxicity, it was suggested that a reduction in FKN levels might affect both the cells' transmigration and adhesion and also cytotoxic NK cell‐arbitrated endothelium injury. In pulmonary arterial hypertension, T cells upregulated the exhibition of CX3CR1 elevated plasma FKN concentrations, and increased FKN mRNA and protein product in pulmonary artery endothelial cells were brought to observation.^89^

FKN is profusely expressed on endothelial cells in skin lesions in atopic dermatitis patients. Serum soluble FKN levels and the quantity of CX3CR1‐positive cells were upregulated and were related to the severity of the disease and decreased after the skin lesions recovery.^90^ This indicates that the FKN system performs an essential function in the CX3CR1‐positive cell trafficking during inflammation.

A previous study revealed that the reduction in serum FKN levels subsequent to prostaglandin E1 (PGE1) therapy might result from the inhibition of FKN expression in human endothelial cells, as demonstrated for 15‐deoxy‐D12, 14‐prostaglandin J2.^91^

## 4 CX3CL1/CX3CR1 IN FIBROSIS (NORMAL AND SSC)

Fibrosis is the hardening and overgrowth, and/or scarring of different tissues that is predominantly mediated via fibroblasts.^92^ Fibrosis is an intricate course involving various chemokines and cytokines that crosstalks with effector cells. CX3CR1/FKN pathway takes on a notable part of the induction and maintenance of fibrosis, possibly through varying mechanisms. It plays a possible role in fibrosis induction through not only direct impacts on fibroblasts but also indirect impacts mediated by the release of cytokine from CX3CR1 leukocytes and macrophages in the lesional tissues. Previous studies reported that raised FKN levels in sera were correlated with pulmonary fibrosis severity in patients with SSc.^20^, ^82^

The disturbance of the interaction between CX3CR1 and FKN has been revealed to suppress the process of fibrosis in a murine model of cytokine‐induced SSc.

Relatively high heterogeneity in the expression of CX3CR1 via NK cells was observed in SSc patients, and in addition, a positive alliance with the anti‐topoisomerase1 antibodies and pulmonary fibrosis was also conducted.^10^

A recent study claimed that modified Rodnan skin scores were positively compatible with FKN serum levels. Furthermore, skin ulcers and digital ischemia were observed to be less frequent in patients with normal serum levels of FKN than in those with elevated FKN.^29^ Immunofluorescence staining confirmed the expression of both CX3CL1 and CX3CR1 on healthy human skin fibroblast cell lines. The expression of CX3CL1 and CX3CR1 was augmented by TGFβ1 stimulation.^11^

Transforming growth factor‐beta (TGF‐β) plays a vital role in tissue repairing and wound healing by stimulating the production of a variety of extracellular matrix proteins.^93^ TGF‐β plays an important role in fibrogenesis by utilizing signaling molecules like Smad2 and Smad3.^94^ Daily injection of subcutaneous bleomycin triggered the expression of skin TGF‐b1 and phosphorylated Smad3 and elevated lesion and serum CX3CL1 expression. Anti‐CX3CL1 monoclonal antibody therapy impeded Smad3 phosphorylation and fibronectin1 and collagen I expression in TGF‐β1 stimulated dermal fibroblasts. CX3CR1 deficiency or Anti‐CX3CL1m antibody remarkably attenuated the fibrosis, vascular injury, and skin inflammation led by bleomycin. In another model, anti‐CX3CL1mAb noticeably declined the skin fibrosis urged by connective tissue growth factor and TGF‐b serial subcutaneous injection.^95^

Healthy lung fibroblasts stimulated with IFN‐γ and IL‐1β, secret FKN, cause pulmonary inflammation and fibrosis.^96^, ^97^ Previous studies depicted that FKN concentration was elevated in SSc patients' lung tissues.^20^, ^98^ Hasegawa et al. declared that CX3CL1 and CX3CR1 were notably escalated in pulmonary fibrosis patients compared to those without pCX3CR1lmonary evidence by HRCT evaluation, which is because of the recruitment of CX3CR1‐positive cells to the lungs. Moreover, the forced vital capacity percentage (FVC%) of the lungs was negatively associated with serum FKN.^20^ Notwithstanding that the part of FKN in the lung fibrosis pathogenesis abides ambiguous, lung fibrosis induced by bleomycin in mice was correlated with upregulated expression of CX3CR1 on M2‐type macrophages and fibroblasts, which played a central part in fibrosis. Bleomycin‐induced fibrosis was diminished in CX3CR1^‐/‐^ mice by suppressing the infiltration of M2 macrophage and fibroblasts.^77^ Additionally, a reduction of fibrosis in other organs, induced by CX3CR1 deficiency, was also observed in concern with FKN.^99^, ^100^, ^101^, ^102^

In SSc mouse models, the axis blockade of CX3CL1‐CX3CR1 can proficiently improve vascular injury and fibrosis transpiring after skin inflammation. Anti‐CX3CL1mAb therapy could be considered a new approach for inflammation‐driven fibrotic skin defects like SSc.^95^

Recent studies have shown that in vitro CX3CL1 contributes to the increased proliferation of cardio fibroblasts and their collagen synthesis.^103^, ^104^

Over a decade ago, disease‐modifying anti‐rheumatic drugs (DMARDs) such as Methotrexate (MTX) were considered to be of limited use in SSc, and the only proven efficacy was the use of MTX for SSc patients suffering from skin thickening.^105^ Multiple studies have been conducted on the role of MTX as an alternative therapeutic option to SSc. MTX's efficacy was analyzed in a 24‐week randomized double‐control trial, and 15 mg of weekly doses were given to patients. Most patients' total skin scores significantly improved after receiving MTX. Several patients with poor response to the initial 15 mg dose of MTX later on had the desired response with 25 mg of weekly MTX.^106^ On the contrary, Krishna et al. concluded that a 6‐month treatment with 15 mg of weekly MTX was only successful in the subjective improvement and mouth opening, but skin score and lung function did not improve significantly.^107^ Seyger et al. assessed the histological change after MTX therapy in patients with localized and systemic sclerosis. It was observed that although significant improvement in both groups was seen after 24 weeks of receiving 15 mg weekly doses of MTX, no considerable change in the histological pattern of skin biopsies and epidermal proliferation in systemic sclerosis was observed. However, localized sclerosis biopsies demonstrated significant alleviation in tenascin staining and mast cell numbers.^108^

E6011, an anti‐FKN monoclonal antibody, is a new emerging drug that has been put into assessment in patients with rheumatoid diseases.^109^ Tanaka et al. assessed the efficacy and safety of this humanized IgG2 monoclonal antibody in RA patients who had inadequate response to Methotrexate treatment. The results demonstrated an excellent response to treatment after 24 weeks of E6011 therapy, especially in patients with higher proportions of CD16+ monocytes at baseline in contrast to 12 weeks of treatment.^110^, ^111^ Moreover, Tanaka et al. also observed the efficacy of this anti‐FKN in RA patients.^112^ In addition, it was also observed that individuals with Crohn's disease benefited from this drug.^113^ Furthermore, it can be derived that the usual MTX doses, 15 mg/weekly, do not strongly affect reducing FKN levels. There is a lack of data on utilizing higher doses of MTX in systemic sclerosis and its impact on histological patterns, and this calls for further assessment of the impact of high doses of MTX on SSc patients.

## 5 CX3CL1/CX3CR1 IN VASCULITIS (NORMAL AND SSC)

Vasculitis is a clinicopathological course distinguished by necrosis and inflammation of vessels, which will lead to occlusion and cause ischemia in the vessels' supplementary end organs. The advances toward a better understanding of vasculitis pathology have highlighted the mediator role of adhesion molecules and cytokines in the interactions between endothelial cells and leukocytes, which defines inflammatory responses. The most critical adhesion molecules are the immunoglobulin superfamily, the intern family, and the selectin family. IL‐1 AND TNF‐α are the two particular cytokines involved in vasculitis's pathogenesis. The mentioned cytokines cause some biological effects, such as the promotion of leukocyte activation, adhesion, and migration through inducing endothelial cells to express adhesion molecules or the inducing the endothelial cells to secrete IL‐8 and IL‐6, which in turn will facilitate the local stimulation of leukocytes (B cells, T cells, and neutrophils) and the neighboring vascular endothelial cells.^114^

The endothelium is the first impediment to the transmigration of leukocytes, acting as a gateway that regulates the extravasation of leukocytes at the site of inflammation. ECs substantiate the secretion and expression of adhesion molecules, numerous cytokines, chemokines, and other elements participating in systemic vasculitis pathogenesis.^115^, ^116^

TNF‐α and IL‐6 are pro‐inflammatory cytokines synthesized and released by neutrophils, monocytes, and mast cells.^117^, ^118^ Morimura et al. observed that TNF‐α and IL‐6 were reduced in CX3CR1^‐/‐^ altered mice and this cytokines' decline matched the number of mast cells and neutrophils that infiltrated the inflammation site.^119^ These outcomes collectively suggested that CX3CR1 has a role in the mast cells and neutrophils infiltration that affects cytokine production. TNF‐α produced by monocytes, mast cells, and neutrophils is observed to prompt expression of CX3CL1 in ECs and, therefore, will enhance immune cell migration via CX3CL1–CX3CR1 interaction.^120^

CX3CL1, expressed in the ECs, can operate as a CX3CR1‐positive leukocyte vascular gatekeeper through swift cell capturing from the blood and enhancing tissue extravasation. The adhesion of Von Willebrand (VWF) glycoprotein to platelets is raised in the CX3CL1 presence.^121^ Increased adhesion of activated ECs to platelet in vitro and unscathed human arteries in a CX3CL1‐dependent manner propose that expression of CX3CL1 via ECs, accompanied by von Willebrand factor, is in concern with the development of vasculopathy‐like atherosclerosis.^119^

Elevated expression of CX3CL1 has been obtained in different vasculitic and autoimmune disorders like RA and systemic lupus erythematosus (SLE), possibly playing a role in neuropsychiatric manifestations and synovitis.^23^ An animal model that underwent studies has depicted that inhibition of CX3CL1 could detain the institution and progressions of lupus nephritis and reduce vasculitis and glomerulosclerosis in MRL/*lpr* mice.^122^

It is found that the raised level of FKN corresponds conclusively with the level of C‐reactive protein (CRP), erythrocyte sedimentation rate (ESR), and vasculitis disease activity in microscopic polyangiitis patients and every systemic vasculitis patient.^29^

Lately, Kasama et al. announced that unregulated expression of CX3CR1 on endothelial cells and the amassing of activated inflammatory cells could expectedly epitomize pathophysiologic circumstances causing skin vasculitis.^29^, ^87^ However, in another study, no significant perivascular or vascular CX3CL1 staining from adjacent vessels with or without vasculopathy was observed.^98^

Lucas et al. noted the function of FKN in vascular remodeling by using smooth muscle cells, an eminent histological feature of both SSc and pulmonary arterial hypertension (PAH). Moreover, oxygen levels regulate the remodeling by smooth muscle mitogens and expression of vasoactive substances, in addition to intervening with angiogenesis mechanisms.^123^ SSc is accompanied by dysregulated angiogenesis due to striking abnormalities in the circulating regulators of angiogenesis, such as FGF‐2, PDGF, VEGF, and PlGF.^124^ FKN plays a notable part in facilitating inflammatory angiogenesis via activating the signal pathways of the phosphatidylinositol 3‐kinase (PI3K)/Akt/eNOS and the G protein‐coupled receptor‐mediated Raf‐1/MEK/ERK.^125^ Enthrallingly, inhibition of FKN expression while functioning as an angiogenic mediator can be induced by hypoxia. Consequently, it contributes to either the pulmonary arterial lesion of PAH or altered angiogenesis and the ischemic changes of SSc vasculopathy. However, the fundamental mechanism for the formation of atherosclerosis is probably dissimilar from those conducting the vascular remodeling perceived in PAH and SSc, where pathological similarities might offer similar pathogenesis of tissue injury.^25^

In patients with SSc, vasculopathy generally goes in advance of tissue fibrosis. Vasculopathy is distinguished by diminished vasculogenesis and angiogenesis, and the perseverance of these impairments sequels in the functional loss of blood flow and capillaries.^126^ Consequently, dermal capillaries' density and structure in bleomycin‐injected murine models were evaluated. Even though bleomycin therapy noticeably decreased the capillary quantity, concurrent anti‐mouse CX3CL1mAb therapy averted this microvascular injury. Endothelial cells' capability of transformation to mesenchymal cells(endothelial to mesenchymal transition, in which endothelial cells acquire myofibroblast‐like characteristics and lose their particular morphology markers) has been established, and this could provide an evaluating source for the fibrotic process.^127^ Furthermore, this transition can cause the loss of endothelial cells through fibrosis induction. Lately, TGFβ‐induced endothelial to mesenchymal transition has been perceived in SSc mouse models and patients with SSc.^128^ Conclusively, even though the part of the CX3CL1/CX3CR1 axis in the transition of endothelial to mesenchymal is left a mystery, this data proposes that anti‐CX3CL1mAb treatment could be convenient for sheltering against vascular injury during SSc.^11^

## 6 CONCLUSION

Emerging evidence claims that FKN could be expressed in numerous tissues, taking part in the CX3CR1‐positive cell accumulation at sites of inflammation and, as a result, be involved in multiple rheumatic diseases such as RA, SLE, and scleroderma.^31^ These data have helped make FKN an appealing therapeutic target. To achieve highly targeted therapeutic approaches, a more advantageous comprehension of the FKN's synthesis, shedding, and cell‐specific activation is required.^9^ The correlation between increased CX3CL1 levels in SSc‐interstitial lung disease (ILD) was established. The ILD prediction and its progression may be available through augmented concentrations in SSc patients.^129^

According to a recent review conducted on the emerging role of FKN in treating rheumatoid diseases, E6011 was observed to subdue a significant contributor to inflammation and alleviate collateral fibrotic and cardiovascular diseases, which, in turn, makes E6011 a novel interventional approach to treating rheumatoid diseases.^130^ Therefore, alleviating FKN levels was conducted to be of significant worth in treating rheumatoid diseases, and this discovery can lead medicine to try new therapeutic approaches to aim at FKL in treating SSc.

Conclusively, serum FKN may play a prominent part in the SSc pathogenesis, involving vascular injury and tissue inflammation. Its estimation could be used as a serological marker for the recognition and follow‐up of skin and pulmonary complications.^29^

## AUTHOR CONTRIBUTIONS

**Fatemehsadat Pezeshkian**: Conceptualization; data curation; investigation; project administration; visualization; writing—original draft; writing—review and editing. **Reza Shahriarirad**: Conceptualization; investigation; methodology; supervision; writing—review and editing. **Hadiseh Mahram**: Data curation; investigation; project administration; writing—original draft; writing—review and editing.

## CONFLICT OF INTEREST STATEMENT

The authors declare no conflict of interest.

## ETHICS STATEMENT

The present study was approved by the Medical Ethics Committee of Shiraz University of Medical Sciences. Obtaining consent was not applicable based on the nature of the study.

## Data Availability

All data regarding this report has been reported in the manuscript. Please contact the corresponding author in case of requiring any further information.

## References

[1] Gabrielli A , Avvedimento EV , Krieg T . Scleroderma. N Engl J Med. 2009;360(19):1989‐2003. 19420368 10.1056/NEJMra0806188

[2] Frech T , Hatton N , Markewitz B , et al. The vascular microenvironment and systemic sclerosis. Int J Rheumatol. 2010;2010:1‐6. 10.1155/2010/362868 PMC2931393 20814552

[3] Clements P , Medsger Jr. T , Feghali C . Cutaneous involvement in systemic sclerosis. Systemic sclerosis Philadelphia. Lippincott Williams & Wilkins; 2004:129‐150.

[4] Denton CP , Black CM . Targeted therapy comes of age in scleroderma. Trends Immunol. 2005;26(11):596‐602. 16168710 10.1016/j.it.2005.09.002

[5] Charles C , Clements P , Furst DE . Systemic sclerosis: hypothesis‐driven treatment strategies. Lancet. 2006;367(9523):1683‐1691. 16714190 10.1016/S0140-6736(06)68737-0

[6] Varga J , Abraham D . Systemic sclerosis: a prototypic multisystem fibrotic disorder. J Clin Invest. 2007;117(3):557‐567. 17332883 10.1172/JCI31139 PMC1804347

[7] Varga JA , Trojanowska M . Fibrosis in systemic sclerosis. Rheum Dis Clin North America. 2008;34(1):115‐143. 10.1016/j.rdc.2007.11.002 PMC9904084 18329536

[8] Kahaleh MB . Vascular involvement in systemic sclerosis (SSc). Clin Exp Rheumatol. 2004;22(3 suppl 33):19‐23. 15344592

[9] Jones B , Koch AE , Ahmed S . Pathological role of fractalkine/CX3CL1 in rheumatic diseases: a unique chemokine with multiple functions. Front Immunol. 2011;2:82. 22566871 10.3389/fimmu.2011.00082 PMC3341950

[10] Benyamine A , Magalon J , Sabatier F , et al. Natural killer cells exhibit a peculiar phenotypic profile in systemic sclerosis and are potent inducers of endothelial microparticles release. Front Immunol. 2018;9:1665. 30072999 10.3389/fimmu.2018.01665 PMC6058015

[11] Luong VH , Utsunomiya A , Chino T , et al. Inhibition of the progression of skin inflammation, fibrosis, and vascular injury by blockade of the CX3CL1/CX3CR1 pathway in experimental mouse models of systemic sclerosis. Arthritis Rheum. 2019;71(11):1923‐1934. 10.1002/art.41009 31173491

[12] Geissmann F , Jung S , Littman DR . Blood monocytes consist of two principal subsets with distinct migratory properties. Immunity. 2003;19(1):71‐82. 12871640 10.1016/s1074-7613(03)00174-2

[13] Del Papa N , Pignataro F . The role of endothelial progenitors in the repair of vascular damage in systemic sclerosis. Front Immunol. 2018;9:1383. 29967618 10.3389/fimmu.2018.01383 PMC6015881

[14] Rossi P , Granel B , Marziale D , Le Mée F , Francès Y . Endothelial function and hemodynamics in systemic sclerosis. Clin Physiol Funct Imaging. 2010;30(6):453‐459. 20718808 10.1111/j.1475-097X.2010.00965.x

[15] LeRoy EC , Black C , Fleischmajer R , et al. Scleroderma (systemic sclerosis): classification, subsets and pathogenesis. J Rheumatol. 1988;15(2):202‐205. 3361530

[16] Matucci‐Cerinic M , Kahaleh B , Wigley FM . Evidence that systemic sclerosis is a vascular disease. Arthrit Rheumat. 2013;65(8):1953‐1962. 10.1002/art.37988 23666787

[17] Blann AD , Woywodt A , Bertolini F , et al. Circulating endothelial cells. Biomarker of vascular disease. Thromb Haemost. 2005;93(2):228‐235. 15711737 10.1160/TH04-09-0578

[18] Chironi GN , Boulanger CM , Simon A , Dignat‐George F , Freyssinet JM , Tedgui A . Endothelial microparticles in diseases. Cell Tissue Res. 2009;335(1):143‐151. 18989704 10.1007/s00441-008-0710-9

[19] Kahaleh MB . Endothelin, an endothelial‐dependent vasoconstrictor in scleroderma. Enhanced production and profibrotic action. Arthritis Rheum. 1991;34(8):978‐983. 1859492 10.1002/art.1780340807

[20] Hasegawa M . Up regulated expression of fractalkine/CX3CL1 and CX3CR1 in patients with systemic sclerosis. Ann Rheum Dis. 2005;64(1):21‐28. 15608300 10.1136/ard.2003.018705 PMC1755178

[21] Umehara H , Bloom ET , Okazaki T , Nagano Y , Yoshie O , Imai T . Fractalkine in vascular biology: from basic research to clinical disease. Arterioscler Thromb Vasc Biol. 2004;24(1):34‐40. 12969992 10.1161/01.ATV.0000095360.62479.1F

[22] Bazan JF , Bacon KB , Hardiman G , et al. A new class of membrane‐bound chemokine with a CX 3 C motif. Nature. 1997;385(6617):640‐644. 9024663 10.1038/385640a0

[23] Yajima N , Kasama T , Isozaki T , et al. Elevated levels of soluble fractalkine in active systemic lupus erythematosus: potential involvement in neuropsychiatric manifestations. Arthritis Rheumat. 2005;52(6):1670‐1675. 15934075 10.1002/art.21042

[24] Bjerkeli V , Damas JK , Fevang B , Holter JC , Aukrust P , Froland SS . Increased expression of fractalkine (CX3CL1) and its receptor, CX3CR1, in Wegener's granulomatosis—possible role in vascular inflammation. Rheumatology. 2007;46(9):1422‐1427. 17616549 10.1093/rheumatology/kem168

[25] Marasini B , Cossutta R , Selmi C , et al. Polymorphism of the fractalkine receptor CX3CR1 and systemic sclerosis‐associated pulmonary arterial hypertension. Clin Dev Immunol. 2005;12:275‐279. 16584113 10.1080/17402520500303297 PMC2270742

[26] Benyamine A , Magalon J , Cointe S , et al. Increased serum levels of fractalkine and mobilisation of CD34+CD45− endothelial progenitor cells in systemic sclerosis. Arthritis Res Ther. 2017;19(1):60. 28320472 10.1186/s13075-017-1271-7 PMC5359964

[27] El‐Sergany M , Shahba A , Ghazy M , et al. Increased expression of soluble fractalkine (CX3CL1) in systemic sclerosis – possible role in vascular inflammation. Egyptian Rheumatol. 2011;33(2):93‐98.

[28] Abraham DJ , Krieg T , Distler J , Distler O . Overview of pathogenesis of systemic sclerosis. Rheumatology. 2006;48(suppl 3):iii3‐iii7. 10.1093/rheumatology/ken481 19487220

[29] El‐Sergany M , Shahba A , Ghazy M , et al. Increased expression of soluble fractalkine (CX3CL1) in systemic sclerosis–possible role in vascular inflammation. Egypt Rheumatol. 2011;33(2):93‐98.

[30] Arai M , Ikawa Y , Chujo S , et al. Chemokine receptors CCR2 and CX3CR1 regulate skin fibrosis in the mouse model of cytokine‐induced systemic sclerosis. J Dermatol Sci. 2013;69(3):250‐258. 23142052 10.1016/j.jdermsci.2012.10.010

[31] Umehara H , Tanaka M , Sawaki T , et al. Fractalkine in rheumatoid arthritis and allied conditions. Modern Rheumatol. 2006;16(3):124‐130. 10.1007/s10165-006-0471-9 16767549

[32] Andréasson K , Alrawi Z , Persson A , Jönsson G , Marsal J . Intestinal dysbiosis is common in systemic sclerosis and associated with gastrointestinal and extraintestinal features of disease. Arthritis Res Ther. 2016;18:278. 27894337 10.1186/s13075-016-1182-z PMC5126986

[33] Niess JH , Brand S , Gu X , et al. CX3CR1‐mediated dendritic cell access to the intestinal lumen and bacterial clearance. Science. 2005;307(5707):254‐258. 15653504 10.1126/science.1102901

[34] Estaleen RA , Reilly CM , Luo XM . A double‐edged sword: interactions of CX3CL1/CX3CR1 and gut microbiota in systemic lupus erythematosus. Front Immunol. 2024;14:1330500. 38299151 10.3389/fimmu.2023.1330500 PMC10828040

[35] Mosser DM , Edwards JP . Exploring the full spectrum of macrophage activation. Nat Rev Immunol. 2008;8(12):958‐969. 19029990 10.1038/nri2448 PMC2724991

[36] Sunderkötter C , Nikolic T , Dillon MJ , et al. Subpopulations of mouse blood monocytes differ in maturation stage and inflammatory response. J Immunology. 2004;172(7):4410‐4417. 15034056 10.4049/jimmunol.172.7.4410

[37] Baggiolini M , Dewald B , Moser B . Interleukin‐8 and related chemotactic cytokines‐CXC and CC chemokines. Adv Immunol. 1994;55:97‐179. 8304236

[38] Murphy PM . The molecular biology of leukocyte chemoattractant receptors. Annu Rev Immunol. 1994;12(1):593‐633. 8011292 10.1146/annurev.iy.12.040194.003113

[39] Rollins BJ . Chemokines. Blood. 1997;90(3):909‐928. 9242519

[40] Biber K , Neumann H , Inoue K , Boddeke HWGM . Neuronal ‘on’ and ‘off' signals control microglia. Trends Neurosci. 2007;30(11):596‐602. 17950926 10.1016/j.tins.2007.08.007

[41] Rogers JT , Morganti JM , Bachstetter AD , et al. CX3CR1 deficiency leads to impairment of hippocampal cognitive function and synaptic plasticity. J Neurosci. 2011;31(45):16241‐16250. 22072675 10.1523/JNEUROSCI.3667-11.2011 PMC3236509

[42] Casas C , Manzano R , Vaz R , Osta R , Brites D . Synaptic failure: focus in an integrative view of ALS. Brain Plasticity. 2016;1(2):159‐175. 29765840 10.3233/BPL-140001 PMC5928542

[43] Hoek RM , Ruuls SR , Murphy CA , et al. Down‐regulation of the macrophage lineage through interaction with OX2 (CD200). Science. 2000;290(5497):1768‐1771. 11099416 10.1126/science.290.5497.1768

[44] Cardona AE , Pioro EP , Sasse ME , et al. Control of microglial neurotoxicity by the fractalkine receptor. Nature Neurosci. 2006;9(7):917‐924. 16732273 10.1038/nn1715

[45] Colafrancesco S , Priori R , Astorri E , et al. FRI0500 evaluation of the role of fractalkine chemokine CX3CL1 and its receptor CX3CR1 in inflammatory myopathies. Ann Rheum Dis. 2014;73(suppl 2):568.

[46] Gerard C , Rollins BJ . Chemokines and disease. Nature Immunol. 2001;2:108‐115. 11175802 10.1038/84209

[47] Proudfoot AEI . Chemokine receptors: multifaceted therapeutic targets. Nat Rev Immunol. 2002;2(2):106‐115. 11910892 10.1038/nri722 PMC7097668

[48] Bazan JF , Bacon KB , Hardiman G , et al. A new class of membrane‐bound chemokine with a CX3C motif. Nature. 1997;385(6617):640‐644. 9024663 10.1038/385640a0

[49] Pan Y , Lloyd C , Zhou H , et al. Neurotactin, a membrane‐anchored chemokine upregulated in brain inflammation. Nature. 1997;387(6633):611‐617. 9177350 10.1038/42491

[50] Umehara H , Bloom E , Okazaki T , Domae N , Imai T . Fractalkine and vascular injury. Trends Immunol. 2001;22(11):602‐607. 11698220 10.1016/s1471-4906(01)02051-8

[51] Kim K‐W , Vallon‐Eberhard A , Zigmond E , et al. In vivo structure/function and expression analysis of the CX3C chemokine fractalkine. Blood. 2011;118(22):e156‐e167. 21951685 10.1182/blood-2011-04-348946 PMC4507037

[52] Fraticelli P , Sironi M , Bianchi G , et al. Fractalkine (CX3CL1) as an amplification circuit of polarized Th1 responses. J Clin Invest. 2001;107(9):1173‐1181. 11342581 10.1172/JCI11517 PMC209276

[53] Cupovic J , Onder L , Gil‐Cruz C , et al. Central nervous system stromal cells control local CD8(+) T cell responses during virus‐induced neuroinflammation. Immunity. 2016;44(3):622‐633. 26921107 10.1016/j.immuni.2015.12.022 PMC7111064

[54] Nomiyama H , Osada N , Yoshie O . The evolution of mammalian chemokine genes. Cytokine Growth Factor Rev. 2010;21(4):253‐262. 20434943 10.1016/j.cytogfr.2010.03.004

[55] Imai T , Hieshima K , Haskell C , et al. Identification and molecular characterization of fractalkine receptor CX3CR1, which mediates both leukocyte migration and adhesion. Cell. 1997;91(4):521‐530. 9390561 10.1016/s0092-8674(00)80438-9

[56] Yoshie O , Imai T , Nomiyama H . Chemokines in immunity. Advances in immunology. 78. Elsevier; 2001:57‐110. 11432208 10.1016/s0065-2776(01)78002-9

[57] Italiani P , Boraschi D . From monocytes to M1/M2 macrophages: phenotypical vs. functional differentiation. Front Immunol. 2014;5:514. 25368618 10.3389/fimmu.2014.00514 PMC4201108

[58] Nishimura M , Umehara H , Nakayama T , et al. Dual functions of fractalkine/CX3C ligand 1 in trafficking of perforin+/granzyme B+ cytotoxic effector lymphocytes that are defined by CX3CR1 expression. J Immunol. 2002;168(12):6173‐6180. 12055230 10.4049/jimmunol.168.12.6173

[59] Garton KJ , Gough PJ , Blobel CP , et al. Tumor necrosis Factor‐α‐converting enzyme (ADAM17) mediates the cleavage and shedding of fractalkine (CX3CL1). J Biol Chem. 2001;276(41):37993‐38001. 11495925 10.1074/jbc.M106434200

[60] Tsou CL , Haskell CA , Charo IF . Tumor necrosis factor‐α‐converting enzyme mediates the inducible cleavage of fractalkine. J Biol Chem. 2001;276(48):44622‐44626. 11571300 10.1074/jbc.M107327200

[61] Fong AM , Robinson LA , Steeber DA , et al. Fractalkine and CX3CR1 mediate a novel mechanism of leukocyte capture, firm adhesion, and activation under physiologic flow. J Exp Med. 1998;188(8):1413‐1419. 9782118 10.1084/jem.188.8.1413 PMC2213407

[62] Hundhausen C , Misztela D , Berkhout TA , et al. The disintegrin‐like metalloproteinase ADAM10 is involved in constitutive cleavage of CX3CL1 (fractalkine) and regulates CX3CL1‐mediated cell‐cell adhesion. Blood. 2003;102(4):1186‐1195. 12714508 10.1182/blood-2002-12-3775

[63] Goda S , Imai T , Yoshie O , et al. CX3C‐chemokine, fractalkine‐enhanced adhesion of THP‐1 cells to endothelial cells through integrin‐dependent and‐independent mechanisms. J Immunol. 2000;164(8):4313‐4320. 10754331 10.4049/jimmunol.164.8.4313

[64] Haskell CA , Cleary MD , Charo IF . Molecular uncoupling of fractalkine‐mediated cell adhesion and signal transduction. J Biol Chem. 1999;274(15):10053‐10058. 10187784 10.1074/jbc.274.15.10053

[65] Umehara H , Goda S , Imai T , et al. Fractalkine, a CX3C‐chemokine, functions predominantly as an adhesion molecule in monocytic cell line THP‐1. Immunol Cell Biol. 2001;79(3):298‐302. 11380684 10.1046/j.1440-1711.2001.01004.x

[66] Turner SL , Mangnall D , Bird NC , Blair‐Zajdel ME , Bunning RA . Effects of pro‐inflammatory cytokines on the production of soluble fractalkine and ADAM17 by HepG2 cells. J Gastrointest Liver Dis. 2010;19(3):265‐271. 20922190

[67] Düsterhöft S , Lokau J , Garbers C . The metalloprotease ADAM17 in inflammation and cancer. Pathology Res Pract. 2019;215(6):152410. 10.1016/j.prp.2019.04.002 30992230

[68] Muraoka S , Nishio J , Kuboi Y , Imai T , Nanki T . Rationale for and clinical development of anti‐fractalkine antibody in rheumatic diseases. Expert Opin Biol Ther. 2020;20:1‐11. 32401060 10.1080/14712598.2020.1764931

[69] Ziegler‐Heitbrock L , Ancuta P , Crowe S , et al. Nomenclature of monocytes and dendritic cells in blood. Blood. 2010;116(16):e74‐e80. 20628149 10.1182/blood-2010-02-258558

[70] Ancuta P , Rao R , Moses A , et al. Fractalkine preferentially mediates arrest and migration of CD16+ monocytes. J Exp Med. 2003;197(12):1701‐1707. 12810688 10.1084/jem.20022156 PMC2193954

[71] Wong KL , Tai JJY , Wong WC , et al. Gene expression profiling reveals the defining features of the classical, intermediate, and nonclassical human monocyte subsets. Blood. 2011;118(5):e16‐e31. 21653326 10.1182/blood-2010-12-326355

[72] Kawamura SOT . Monopoiesis in humans and mice. Int Immunol. 2018;30:503‐509. 30247712 10.1093/intimm/dxy063

[73] Volin MV , Woods JM , Amin MA , Connors MA , Harlow LA , Koch AE . Fractalkine: a novel angiogenic chemokine in rheumatoid arthritis. Am J Pathol. 2001;159(4):1521‐1530. 11583978 10.1016/S0002-9440(10)62537-0 PMC1850492

[74] !!! INVALID CITATION!!! (71, 72,73).

[75] Matucci‐Cerinic M , Kahaleh M , LeRoy E . Pathogenesis: Vascular Involvement. Systemic Sclerosis Baltimore. Williams & Wilkins; 1996:153‐174.

[76] Kahaleh MB , Fan PS . Mechanism of serum‐mediated endothelial injury in scleroderma: identification of a granular enzyme in scleroderma skin and sera. Clin Immunol Immunopathol. 1997;83(1):32‐40. 9073533 10.1006/clin.1996.4322

[77] Ishida Y , Kimura A , Nosaka M , et al. Essential involvement of the CX3CL1‐CX3CR1 axis in bleomycin‐induced pulmonary fibrosis via regulation of fibrocyte and M2 macrophage migration. Sci Rep. 2017;7(1):16833. 29203799 10.1038/s41598-017-17007-8 PMC5714949

[78] Lionakis MS , Swamydas M , Fischer BG , et al. CX 3 CR1‐dependent renal macrophage survival promotes Candida control and host survival. J Clin Invest. 2013;123(12):5035‐5051. 24177428 10.1172/JCI71307 PMC3859390

[79] White GE , Greaves DR . Fractalkine: a survivor's guide: chemokines as antiapoptotic mediators. Arterioscler Thromb Vasc Biol. 2012;32(3):589‐594. 22247260 10.1161/ATVBAHA.111.237412

[80] Schwarz N , Pruessmeyer J , Hess FM , et al. Requirements for leukocyte transmigration via the transmembrane chemokine CX3CL1. Cell Mol Life Sci. 2010;67(24):4233‐4248. 20559678 10.1007/s00018-010-0433-4 PMC11115548

[81] Lesnik P , Haskell CA , Charo IF . Decreased atherosclerosis in CX3CR1‐/‐ mice reveals a role for fractalkine in atherogenesis. J Clin Invest. 2003;111(3):333‐340. 12569158 10.1172/JCI15555 PMC151849

[82] Benyamine A , Magalon J , Cointe S , et al. Increased serum levels of fractalkine and mobilisation of CD34(+)CD45(‐) endothelial progenitor cells in systemic sclerosis. Arthritis Res Ther. 2017;19(1):60. 28320472 10.1186/s13075-017-1271-7 PMC5359964

[83] Qin W , Li Z , Luo S , Wu R , Pei Z , Huang R . Exogenous fractalkine enhances proliferation of endothelial cells, promotes migration of endothelial progenitor cells and improves neurological deficits in a rat model of ischemic stroke. Neurosci Lett. 2014;569:80‐84. 24704182 10.1016/j.neulet.2014.03.052

[84] Herlea‐Pana O , Yao L , Heuser‐Baker J , et al. Chemokine receptors CXCR2 and CX3CR1 differentially regulate functional responses of bone‐marrow endothelial progenitors during atherosclerotic plaque regression. Cardiovasc Res. 2015;106(2):324‐337. 25765938 10.1093/cvr/cvv111 PMC4481573

[85] Auffray C , Fogg D , Garfa M , et al. Monitoring of blood vessels and tissues by a population of monocytes with patrolling behavior. Science. 2007;317(5838):666‐670. 17673663 10.1126/science.1142883

[86] Carlin LM , Stamatiades EG , Auffray C , et al. Nr4a1‐dependent Ly6Clow monocytes monitor endothelial cells and orchestrate their disposal. Cell. 2013;153(2):362‐375. 23582326 10.1016/j.cell.2013.03.010 PMC3898614

[87] Kasama T , Wakabayashi K , Sato M , Takahashi R , Isozaki T . Relevance of the CX3CL1/fractalkine‐CX3CR1 pathway in vasculitis and vasculopathy. Transl Res. 2010;155(1):20‐26. 20004358 10.1016/j.trsl.2009.08.009

[88] Yoneda O , Imai T , Goda S , et al. Fractalkine‐mediated endothelial cell injury by NK cells. J Immunology. 2000;164(8):4055‐4062. 10754298 10.4049/jimmunol.164.8.4055

[89] Balabanian K , Foussat A , Dorfmüller P , et al. CX3C chemokine fractalkine in pulmonary arterial hypertension. Am J Respir Crit Care Med. 2002;165(10):1419‐1425. 12016106 10.1164/rccm.2106007

[90] Echigo T , Hasegawa M , Shimada Y , Takehara K , Sato S . Expression of fractalkine and its receptor, CX3CR1, in atopic dermatitis: possible contribution to skin inflammation. J Allergy Clin Immunol. 2004;113(5):940‐948. 15131578 10.1016/j.jaci.2004.02.030

[91] Sicinska J , Gorska E , Cicha M , et al. Increased serum fractalkine in systemic sclerosis. down‐regulation by prostaglandin E1. Clin Exp Rheumatol. 2008;26(4):527‐533. 18799080

[92] Wynn T . Cellular and molecular mechanisms of fibrosis. J Pathol. 2008;214(2):199‐210. 18161745 10.1002/path.2277 PMC2693329

[93] Branton MH , Kopp JB . TGF‐β and fibrosis. Microb Infect. 1999;1(15):1349‐1365. 10.1016/s1286-4579(99)00250-6 10611762

[94] Gauldie J , Bonniaud P , Sime P , Ask K , Kolb M . TGF‐β, Smad3 and the process of progressive fibrosis. Biochem Soc Trans. 2007;35(Pt 4):661‐664. 17635115 10.1042/BST0350661

[95] Hasegawa M , Luong VH , Utsunomiya A , et al. LB1141 anti‐mouse CX3CL1 monoclonal antibody therapy in mouse models of systemic sclerosis. J Invest Dermatol. 2019;139(9):B25.

[96] Elias JA , Freundlich B , Kern JA , Rosenbloom J . Cytokine networks in the regulation of inflammation and fibrosis in the lung. Chest. 1990;97(6):1439‐1445. 2112081 10.1378/chest.97.6.1439

[97] Isozaki T , Otsuka K , Sato M , et al. Synergistic induction of CX3CL1 by interleukin‐1β and interferon‐γ in human lung fibroblasts: involvement of signal transducer and activator of transcription 1 signaling pathways. Transl Res. 2011;157(2):64‐70. 21256458 10.1016/j.trsl.2010.11.007

[98] Hoffmann‐Vold AM , Weigt SS , Palchevskiy V , et al. Augmented concentrations of CX3CL1 are associated with interstitial lung disease in systemic sclerosis. PLoS One. 2018;13(11):e0206545. 30457999 10.1371/journal.pone.0206545 PMC6245508

[99] Engel DR , Krause TA , Snelgrove SL , et al. CX3CR1 reduces kidney fibrosis by inhibiting local proliferation of profibrotic macrophages. J Immunol. 2015;194(4):1628‐1638. 25595779 10.4049/jimmunol.1402149

[100] Peng X , Zhang J , Xiao Z , Dong Y , Du J . CX3CL1–CX3CR1 interaction increases the population of Ly6C−CX3CR1hi macrophages contributing to unilateral ureteral obstruction‐induced fibrosis. J Immunol. 2015;195(6):2797‐2805. 26254342 10.4049/jimmunol.1403209

[101] Shimizu K , Furuichi K , Sakai N , et al. Fractalkine and its receptor, CX3CR1, promote hypertensive interstitial fibrosis in the kidney. Hypertension Res. 2011;34(6):747‐752. 10.1038/hr.2011.23 21451526

[102] Wasmuth HE , Zaldivar MM , Berres M‐L , et al. The fractalkine receptor CX3CR1 is involved in liver fibrosis due to chronic hepatitis C infection. J Hepatol. 2008;48(2):208‐215. 18078680 10.1016/j.jhep.2007.09.008

[103] Gu X , Xu J , Yang XP , Peterson E , Harding P . Fractalkine neutralization improves cardiac function after myocardial infarction. Exp Physiol. 2015;100(7):805‐817. 25943588 10.1113/EP085104 PMC4686137

[104] Xuan W , Liao Y , Chen B , et al. Detrimental effect of fractalkine on myocardial ischaemia and heart failure. Cardiovasc Res. 2011;92(3):385‐393. 21840883 10.1093/cvr/cvr221

[105] Blank N . The role of DMARDs in systemic sclerosis therapy. Rheumatology. 2006;45(suppl 3):iii42‐iii44. 16987834 10.1093/rheumatology/kel289

[106] van den Hoogen FHJ , Boerbooms AMT , Swaak AJG , Rasker JJ , van Lier HJJ , van de Putte LBA . Comparison of methotrexate with placebo in the treatment of systemic sclerosis: a 24 week randomized double‐blind trial, followed by a 24 week observational trial. Rheumatology. 1996;35(4):364‐372. 10.1093/rheumatology/35.4.364 8624641

[107] Krishna Sumanth M , Sharma VK , Khaitan BK , Kapoor A , Tejasvi T . Evaluation of oral methotrexate in the treatment of systemic sclerosis. Int J Dermatol. 2007;46(2):218‐223. 17269983 10.1111/j.1365-4632.2007.02887.x

[108] Seyger MMB , van den Hoogen FHJ , van Vlijmen‐Willems IMJJ , van de Kerkhof PCM , J de Jong EMG . Localized and systemic scleroderma show different histological responses to methotrexate therapy. J Pathol. 2001;193(4):511‐516. 11276011 10.1002/1096-9896(2000)9999:9999<::AID-PATH779>3.0.CO;2-8

[109] Tabuchi H , Katsurabara T , Mori M , et al. Pharmacokinetics, pharmacodynamics, and safety of E6011, a novel humanized antifractalkine (CX3CL1) monoclonal antibody: a randomized, double‐blind, placebo‐controlled single‐ascending‐dose study. J Clin Pharmacol. 2019;59(5):688‐701. 30575978 10.1002/jcph.1361

[110] Tanaka Y , Takeuchi T , Yamanaka H , et al. Efficacy and safety of E6011, an anti‐fractalkine monoclonal antibody, in patients with active rheumatoid arthritis with inadequate response to methotrexate: results of a randomized, double‐blind, placebo‐controlled phase II study. Arthrit Rheumatol. 2021;73(4):587‐595. 10.1002/art.41555 PMC8048525 33038062

[111] Tanaka Y , Takeuchi T , Yamanaka H , et al. A phase 2 study of E6011, an anti‐fractalkine monoclonal antibody, in patients with rheumatoid arthritis inadequately responding to biological disease‐modifying antirheumatic drugs. Modern Rheumatol. 2021;31(4):783‐789. 10.1080/14397595.2020.1868675 33427546

[112] Tanaka Y , Takeuchi T , Umehara H , et al. Safety, pharmacokinetics, and efficacy of E6011, an antifractalkine monoclonal antibody, in a first‐in‐patient phase 1/2 study on rheumatoid arthritis. Modern Rheumatol. 2018;28(1):58‐65. 10.1080/14397595.2017.1337056 28681650

[113] Matsuoka K , Naganuma M , Hibi T , et al. Phase 1 study on the safety and efficacy of E6011, antifractalkine antibody, in patients with Crohn's disease. J Gastroenterol Hepatol. 2021;36(8):2180‐2186. 33599356 10.1111/jgh.15463 PMC8451784

[114] Sneller MC , Fauci AS . Pathogenesis of vasculitis syndromes. Med Clin North Am. 1997;81(1):221‐242. 9012762 10.1016/s0025-7125(05)70512-5

[115] Cid MC , Segarra M , Martínez AG , Hernández‐Rodríguez J . Endothelial cells, antineutrophil cytoplasmic antibodies, and cytokines in the pathogenesis of systemic vasculitis. Curr Rheumatol Rep. 2004;6(3):184‐194. 15134597 10.1007/s11926-004-0067-3

[116] Bacon PA . Endothelial cell dysfunction in systemic vasculitis: new developments and therapeutic prospects. Curr Opin Rheumatol. 2005;17(1):49‐55. 15604904 10.1097/01.bor.0000149084.16639.b0

[117] Vassalli P . The pathophysiology of tumor necrosis factors. Annu Rev Immunol. 1992;10(1):411‐452. 1590993 10.1146/annurev.iy.10.040192.002211

[118] Akira S , Taga T , Kishimoto T . Interleukin‐6 in biology and medicine. Adv Immunol. 1993;54:1‐78. 8379461 10.1016/s0065-2776(08)60532-5

[119] Morimura S , Sugaya M , Sato S . Interaction between CX3CL1 and CX3CR1 regulates vasculitis induced by immune complex deposition. Am J Pathol. 2013;182(5):1640‐1647. 23470165 10.1016/j.ajpath.2013.01.023

[120] Ahn SY , Cho CH , Park KG , et al. Tumor necrosis Factor‐α induces fractalkine expression preferentially in arterial endothelial cells and mithramycin A suppresses TNF‐α‐induced fractalkine expression. Am J Pathol. 2004;164(5):1663‐1672. 15111313 10.1016/s0002-9440(10)63725-x PMC1615656

[121] Meyer dos Santos S , Klinkhardt U , Scholich K , et al. The CX3C chemokine fractalkine mediates platelet adhesion via the von Willebrand receptor glycoprotein Ib. Blood. 2011;117(18):4999‐5008. 21398580 10.1182/blood-2011-02-335471

[122] Inoue A , Hasegawa H , Kohno M , et al. Antagonist of fractalkine (CX3CL1) delays the initiation and ameliorates the progression of lupus nephritis in MRL/lpr mice. Arthritis Rheum. 2005;52(5):1522‐1533. 15880599 10.1002/art.21007

[123] Lucas AD , Bursill C , Guzik TJ , Sadowski J , Channon KM , Greaves DR . Smooth muscle cells in human atherosclerotic plaques express the fractalkine receptor CX3CR1 and undergo chemotaxis to the CX3C chemokine fractalkine (CX3CL1). Circulation. 2003;108(20):2498‐2504. 14581400 10.1161/01.CIR.0000097119.57756.EF

[124] Hummers LK , Hall A , Wigley FM , Simons M . Abnormalities in the regulators of angiogenesis in patients with scleroderma. J Rheumatol. 2009;36(3):576‐582. 19228661 10.3899/jrheum.080516 PMC4020014

[125] Lee SJ , Namkoong S , Kim YM , et al. Fractalkine stimulates angiogenesis by activating the Raf‐1/MEK/ERK‐ and PI3K/Akt/eNOS‐dependent signal pathways. Amer J Physiol Heart Circulat Physiol. 2006;291(6):H2836‐H2846. 10.1152/ajpheart.00113.2006 16877565

[126] Distler JHW . Angiogenesis and vasculogenesis in systemic sclerosis. Rheumatology. 2006;45(suppl 3):iii26‐iii27. 16987827 10.1093/rheumatology/kel295

[127] Piera‐Velazquez S , Li Z , Jimenez SA . Role of endothelial‐mesenchymal transition (EndoMT) in the pathogenesis of fibrotic disorders. Am J Pathol. 2011;179(3):1074‐1080. 21763673 10.1016/j.ajpath.2011.06.001 PMC3157273

[128] Manetti M , Romano E , Rosa I , et al. Endothelial‐to‐mesenchymal transition contributes to endothelial dysfunction and dermal fibrosis in systemic sclerosis. Ann Rheum Dis. 2017;76(5):924‐934. 28062404 10.1136/annrheumdis-2016-210229

[129] Hoffmann‐Vold A‐M , Huynh R , Volkmann E , et al. FRI0257 Augmented Concentrations of Cx3cl1 Are Associated with Progressiv Interstitial Lung Disease in Systemic Sclerosis. BMJ Publishing Group Ltd; 2016. 10.1371/journal.pone.0206545 PMC6245508 30457999

[130] Tanaka Y , Hoshino‐Negishi K , Kuboi Y , Tago F , Yasuda N , Imai T . Emerging role of fractalkine in the treatment of rheumatic diseases. Immunotargets Ther. 2020;9:241‐253. 33178636 10.2147/ITT.S277991 PMC7649223

